# An Enhanced Variant Designed From DLP4 Cationic Peptide Against *Staphylococcus aureus* CVCC 546

**DOI:** 10.3389/fmicb.2020.01057

**Published:** 2020-06-05

**Authors:** Bing Li, Na Yang, Xiumin Wang, Ya Hao, Ruoyu Mao, Zhanzhan Li, Zhenlong Wang, Da Teng, Jianhua Wang

**Affiliations:** ^1^Gene Engineering Laboratory, Feed Research Institute, Chinese Academy of Agricultural Sciences, Beijing, China; ^2^Key Laboratory for Feed Biotechnology, Ministry of Agriculture and Rural Affairs, Beijing, China

**Keywords:** insect defensin, peptide design, bioavailability, *Staphylococcus aureus*, antimicrobial mechanism, pharmacodynamics

## Abstract

Insect defensins are promising candidates for the development of potent antimicrobials against antibiotic-resistant *Staphylococcus aureus* (*S. aureus*). An insect defensin, DLP4, isolated from the hemolymph of *Hermetia illucens* larvae, showed low antimicrobial activity against Gram-positive (G^+^) pathogens and high cytotoxicity, which limited its effective therapeutic application. To obtain more potent and low cytotoxicity molecules, a series of peptides was designed based on the DLP4 template by changing the conservative site, secondary structure, charge, or hydrophobicity. Among them, a variant designated as ID13 exhibited strong antibacterial activity at low MIC values of 4–8 μg/mL to G^+^ pathogens (*S. aureus*: 4 μg/mL; *Staphylococcus epidermidis*: 8 μg/mL; *Streptococcus pneumoniae*: 4 μg/mL; *Streptococcus suis*: 4 μg/mL), which were lower than those of DLP4 (*S. aureus*: 16 μg/mL; *S. epidermidis*: 64 μg/mL; *S. pneumoniae*: 32 μg/mL; *S. suis*: 16 μg/mL), and cytotoxicity of ID13 (71.4% viability) was less than that of DLP4 (63.8% viability). ID13 could penetrate and destroy the cell membrane of *S. aureus* CVCC 546, resulting in an increase in potassium ion leakage; it bound to genomic DNA (gDNA) and led to the change of gDNA conformation. After treatment with ID13, perforated, wrinkled, and collapsed *S. aureus* CVCC 546 cells were observed in electron microscopy. Additionally, ID13 killed over 99.99% of *S. aureus* within 1 h, 2 × MIC of ID13 induced a post-antibiotic effect (PAE) of 12.78 ± 0.28 h, and 10 mg/kg ID13 caused a 1.8 log_10_ (CFU/g) (CFU: colony-forming units) reduction of *S. aureus* in infected mouse thigh muscles and a downregulation of TNF-α, IL-6, and IL-10 levels, which were superior to those of DLP4 or vancomycin. These findings indicate that ID13 may be a promising peptide antimicrobial agent for therapeutic application.

## Introduction

Antibiotics play pivotal roles in disease prevention, growth promotion, and production improvement in animal husbandry (Ferber, 2002; Cheng et al., 2014; Van Boeckel et al., 2015). Nevertheless, the long-term use of antibiotics in animal breeding can accelerate the emergence of antibiotic-resistant bacteria (ARB), including *Staphylococcus aureus*, which can cause trauma infection, abscess, cellulitis, mastitis, endometritis, arthritis, septicemia, and sepsis in animals (Fluit, 2012; Foster, 2012; Dan et al., 2019). It has been found that *S. aureus* is resistant to tetracycline, methicillin, erythromycin, clindamycin, ciprofloxacin, and vancomycin (Hiramatsu, 2001; Lai et al., 2018; Pirolo et al., 2019). The interactions among animals, humans, and the environment have also strengthened the spreading of ARB (Cheng et al., 2013; Laxminarayan et al., 2013; Maertens et al., 2019). These factors not only affect the effectiveness of prevention and treatment of animal diseases but also may endanger public safety (Coyne et al., 2016, 2019). Therefore, alternatives to antibiotics have become the spotlights of research in recent years (Lammie and Hughes, 2016; Moravej et al., 2018a).

Antimicrobial peptides (AMPs) are currently under evaluation as an alternative to antibiotics (Wang et al., 2016; da Cunha et al., 2017; Kang et al., 2017). AMPs have been identified in a variety of organisms (Zhang and Gallo, 2016; Bechinger and Gorr, 2017), including insects, the largest population of living organisms on earth, which have great potential as a source of AMPs (Yi et al., 2014; Koehbach, 2017). Within insects, such a class of disulfide-rich peptides is referred to as “insect defensins” most of them consisted of about 40 amino acid (AA) residues, with an N-terminal loop, an α-helix, and an anti-parallel β-sheet cross-linked by three disulfide bonds (CSαβ) (Cornet et al., 1995; Landon et al., 2008) and they have broad-spectrum activity against various Gram-positive bacteria, including methicillin-resistant *S. aureus* (MRSA) (Józefiak and Engberg, 2017). However, most of these peptides have modest antibacterial activity and cytotoxicity, which limit their active implementation in medicine (Barreto-Santamaria et al., 2019). Based on the key CSαβ scaffold, a few defensins such as tenecin 1, Def-AcAA, and plectasin have been designed with an improved activity and reduced cytotoxicity (Ahn et al., 2006; Landon et al., 2008; Zhang et al., 2014). Although both academia and start-ups are continuing their efforts, there are still few AMP-based antibiotics in use (Roncevic et al., 2019).

In our previous study, an insect defensin, DLP4, isolated from the hemolymph of *Hermetia illucens* larvae (Park et al., 2015) showed a sequence identity ranging from 46.5 to 72.5% with the homologous sequences in the NCBI database. It displayed antibacterial activity against *S. aureus* and proved to be failed to induce resistance (Li et al., 2017). However, the low antibacterial activity (16∼64 μg/mL) and relatively high cytotoxicity of DLP4 limit its effective application. In the study, to improve the antibacterial activity and reduce cytotoxicity of DLP4, a series of derivatives were generated by means of AA mutations in the native sequence (Der Torossian Torres and de la Fuente-Nunez, 2019). In addition, bioavailability, antimicrobial mechanism, and pharmacodynamics were studied *in vitro* and in a mouse thigh model infected with *S. aureus*.

## Materials and Methods

### Bacterial Strains and Cell Lines

The bacterial strains *S. aureus* ATCC 43300, *Staphylococcus epidermidis* ATCC 12228, and *Escherichia coli* ATCC 25922 were purchased from American Type Culture Collection (ATCC). *S. aureus* CVCC 546, *Streptococcus pneumoniae* CVCC 2350, *Streptococcus suis* CVCC 3928, *Salmonella pullorum* CVCC 533, *Salmonella enteritidis* CVCC 3377, and *E. coli* K88 were purchased from the China Veterinary Culture Collection Center (CVCC). RAW 264.7 murine macrophages were obtained from Peking Union Medical College.

### Reagents

Mueller–Hinton broth (MHB) and Mueller–Hinton agar (MHA) were obtained from BEIJING AOBOXING BIO-TECH CO., LTD (China). Sodium dodecyl sulfate (SDS), 3-(4,5-dimethylthiazol-2-yl)-2,5-diphenyltetrazolium bromide (MTT), trifluoroethanol (TFE), and propidium iodide (PI) were purchased from Sigma-Aldrich (China). Dulbecco’s modified Eagle medium (DMEM) and fetal bovine serum (FBS) were obtained from Gibco (China).

### Peptide Design, Expression, and Purification

**FIGURE 1:**
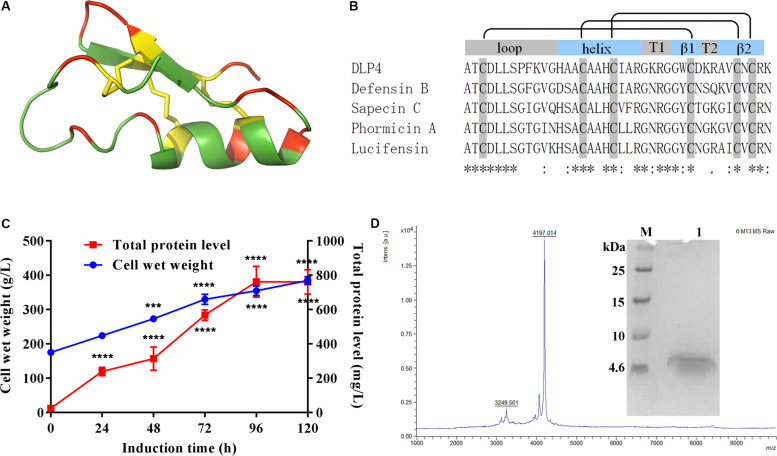
**(A)** Representation of the 3D structure of DLP4 was built from Def-AcAA (PDB: 2nz3). Yellow, red, and green represent cysteines and disulfide bonds and charged and neutrally charged residues, respectively. **(B)** Multiple alignments of the insect defensins derived from APD were carried out using MUSCLE 3.8. The boxes above the alignment represent loop, helix, turns, and β strands, respectively. The black lines indicate the disulfide bond between cysteines. The “*” under the sequences indicates positions which have a single, fully conserved residue, the “:” indicates conservation between groups of strongly similar properties, and the “.” indicates conservation between groups of weakly similar properties. **(C)** Time curves of the total protein levels and cell wet weights. Total protein and biomass are measured every 24 h. Statistical significance of differences between 24 to 120 h and 0 h was determined using the two-way ANOVA and Dunnett’s multiple comparisons. ***p < 0.001; ****p < 0.0001. **(D)** Tricine-SDS-PAGE and MALDI-TOF MS analysis of the purified ID13. M: ultra-low molecular weight protein marker; lane 1: purified ID13.

To develop derivatives of peptide DLP4 with improved efficacy and to study the structure–activity relationship of DLP4, we analyzed sequences containing only six conserved cysteine residues in mature defensins retrieved from Antimicrobial Peptide Database (APD)^1^ and used MUSCLE 3.8 for multiple sequence alignment (Figure 1). Then, a series of derivatives were generated by AA substitution based on the template of parent peptide DLP4.

Recombinant plasmids were constructed by cloning the DLP4 and its analogs encoding sequence into the pPICZαA vector and transformed into *Pichia pastoris* X-33 for expression. Peptide purification was carried out with the AKTAxpress system. The purified peptides were characterized by tricine-sodium dodecyl sulfate polyacrylamide gel electrophoresis (Tricine-SDS-PAGE) and matrix-assisted laser desorption/ionization time-of-flight/time-of-flight tandem mass spectrometer (MALDI-TOF/TOF MS) (Ultraflextreme, Bruker, Germany) (Zhang et al., 2015).

### Structure Determination of Peptides

#### Determination of Disulfide Bonds

The S–S bonds in peptides were characterized through thermolysin enzymolysis followed by MALDI-TOF/TOF MS characterization (Lee et al., 2014). Simply, peptides were digested with thermolysin (Promega, United States) at the peptide/thermolysin ratio of 10:1 (w/w) in 100 mM ammonium acetate, 2 mM CaCl_2_ (pH 6.2) at 60°C for 1 h, after which the hydrolysates were separated by RP-HPLC, and characterized by MALDI-TOF/TOF MS (Ultraflextreme, Bruker, Germany).

#### Circular Dichroism (CD) of Peptides

The secondary structure of AMPs can be determined by CD on a Bio-Logic MOS-450 spectropolarimeter (France). CD measurements of peptides were performed in ddH_2_O, 40 mM SDS, or 50% TFE using a 1.0-mm path-length cuvette; the spectra were recorded from 180 to 260 nm at room temperature three times (Lin et al., 2018).

### Bioavailability Analyses

#### Antimicrobial Activity

The minimal inhibitory concentration (MIC) was evaluated by a microtiter plate assay (Wiegand et al., 2008). Serial 2-fold dilutions of test peptides were placed into a 96-well plate containing identical bacterial inoculum and incubated at 37°C for 16∼20 h. Vancomycin was used as antibiotic control. The MIC value was defined as the lowest peptide concentration where no visible growth occurred. All tests were made in triplicate.

#### Hemolysis

Hemolytic activity of peptides was evaluated with fresh mouse erythrocytes. Briefly, fresh mouse erythrocytes were collected, washed, and resuspended to a concentration of 8% (v/v) in PBS. Then, an aliquot of erythrocyte solution was added to 96-well plates containing an equal volume of peptide solution (1∼256 μg/mL) and was incubated for 1 h at 37°C. The erythrocyte suspension treated with 0.1% Triton X-100 and left untreated was employed as positive and negative controls, respectively. After centrifugation at 2000 rpm for 5 min, the supernatant was measured using a microplate reader at 576 nm. The percent hemolysis was calculated using the following formula: *Percenthemolysis* = [(*A* − *A*_0_)/(*A*_100_ − *A*_0_)] × 100%, where *A*, *A*_100_, and *A*_0_ represent the absorbance of the peptide sample and positive and negative controls, respectively.

#### Mammalian Cytotoxicity

The cytotoxicity of peptides toward mouse macrophage RAW 264.7 was evaluated by the MTT assay as described previously (Wang X. et al., 2018).

### Antimicrobial Mechanism of Peptides

#### Effects of Peptides on *S. aureus* Membrane

##### Membrane permeabilization

The effects of peptides on membrane permeabilization were measured by flow cytometry (Zhu et al., 2018). Briefly, *S. aureus* CVCC 546 cells in the exponential phase (10^8^ CFU/mL, CFU: colony-forming units) were treated with peptides at 1×, 2×, or 4 × MIC and incubated with 50 μL PI at 0.5 mg/mL for 30 min at 25°C. Cells untreated and treated with 2 × MIC DLP4 or vancomycin were used as blank and positive controls. The fluorescence of the dye was monitored at an excitation wavelength of 488 nm and an emission wavelength of 635 nm.

##### Potassium ion (K^+^) leakage

To further investigate the effects of peptides on membrane integrity, K^+^ leakage determination was carried out as previously described (Miao et al., 2016). Briefly, *S. aureus* CVCC 546 cells were centrifuged, washed, and resuspended in 0.9% sterile saline. The cell suspension (10^8^ CFU/mL) was treated with peptides at 1 × MIC. Cells untreated and treated with DLP4 or nisin were used as blank and positive controls. At time intervals of 30, 60, 90, 120, and 150 min, the suspensions were centrifuged at 12000 rpm for 5 min. The supernatants were then subjected to measurement by Agilent inductively coupled plasma optical emission (ICP-OES).

#### Effects of Peptides on *S. aureus* Genomic DNA

##### Gel retardation

A gel retardation assay (Zhao et al., 2019) was utilized to analyze the binding of peptides to genomic DNA (gDNA) extracted from *S. aureus* CVCC 546 with TIANamp Bacteria DNA Kit (TIANGEN Biotech Co., Ltd., Beijing). The peptides were incubated for 10 min with gDNA at different ratios of 0–5.0 (w/w) and then loaded on 1% agarose gel for electrophoresis analysis.

##### Atomic force microscopy (AFM)

Atomic force microscopy images of gDNA-peptide complexes were carried out as described previously (Fojan and Gurevich, 2017). Simply, gDNA (1 μg/mL) was incubated with equal-volume peptides (5 μg/mL) at 30°C for 10 min. Then, the sample was scanned by multimode 8 AFM (Bruker, United States).

##### CD spectra

Circular dichroism measurements (Bakshi et al., 2014) were carried out to examine the effect of peptide on the secondary structure of *S. aureus* CVCC 546 gDNA at peptide/gDNA ratios of 0.5 and 1, respectively. After incubation, the mixtures were loaded and scanned from 220 to 320 nm at room temperature with a Bio-Logic MOS450 spectropolarimeter (France).

### Morphological Observations

To further characterize the bactericidal effects of peptides, scanning electron microscopy (SEM) and transmission electron microscopy (TEM) were used to visualize the morphological changes. *S. aureus* CVCC 546 cells in the exponential phase (10^8^ CFU/mL) were treated with 4 × MIC peptides at 37°C for 2 h or left untreated as control. Cells for SEM or TEM were processed as described previously (Yang et al., 2019) and samples were observed using a QUANTA 200 SEM (FEI, Philips, Netherlands) or a JEM-1400 (JEDL, Tokyo, Japan).

### *In vitro* and *in vivo* Pharmacodynamics

#### Time-Kill Assay

Time-kill assay was used to assess the killing rates of *S. aureus* cells by AMPs *in vitro*, done by measuring the number of viable bacteria left at various times after exposure to the peptides. Simply, reagents were added to MHB medium containing 10^5^ CFU/mL *S. aureus* CVCC 546 at the final concentrations of 1×, 2×, or 4 × MIC; the mixture was incubated at 37°C and 250 rpm. At different time intervals, a certain sample was taken, serially diluted, and plated on MHA for colonies counting. Cells treated with 2 × MIC DLP4 or vancomycin and left without treatment were used as the positive and blank controls, respectively (Flamm et al., 2019).

#### Post-antibiotic Effect (PAE)

The PAE was determined by exposing *S. aureus* cells to antimicrobial agent at 1×, 2×, or 4 × MIC for 2 h. After drug removal by dilution, the bacterial culture was incubated at 37°C and 250 rpm. An aliquot of samples (50 μL) were taken for CFU counts at different time intervals until bacterial cultures became turbid. Bacteria treated with DLP4 or vancomycin and left without treatment were used as the positive and blank controls, respectively. The PAE was calculated using the following formula: *PAE* = *T* − *C*, where *T* is the time for the CFU counts to increase by 10-fold above the count observed immediately after drug removal, and *C* is the corresponding time for the untreated control (Oh et al., 2019).

#### Mouse Thigh Infection Model

Mouse experiments were carried out at Gene Engineering Laboratory, Feed Research Institute of Chinese Academy of Agricultural Sciences (CAAS) and complied with institutional animal care and use policies and procedures (AEC-CAAS-20090609). All animal studies were performed with female CD-1 mice, 6∼7 weeks old.

*S. aureus* CVCC 546 in the exponential phase were resuspended in MHB and adjusted to 1 × 10^8^ CFU/mL, a 0.1-mL inoculum was injected into the right thighs of mice. At 2 h post-infection, mice (*n* = 6) were administered intraperitoneally (i.p.) with peptides at 2.5, 5, or 10 mg/kg. Mice raised without any treatment, or treated with PBS, 10 mg/kg DLP4, or vancomycin were used as the blank and negative and positive controls, respectively. At 24 h post-infection, peripheral blood was collected and serum was separated for cytokine levels analysis by using enzyme-linked immunosorbent assay (ELISA) kit (R&D systems, United States). Mice were euthanized, the right thighs were aseptically removed and processed for colonies counting (Ling et al., 2015).

### Statistical Analysis

All data are presented as mean ± standard deviation (SD), where “*n*” represents the number of animals or samples. Statistical analysis was performed by one-way or two-way analysis of variance (ANOVA) followed by Dunnett’s multiple comparisons test using GraphPad Prism 7 or OriginPro 8. *p* < 0.05 was considered statistically significant.

## Results

### Peptide Design and Expression

As shown in Figures 1A,B, a multiple alignment revealed insect defensins retrieved from APD share high homology of 59%∼87.5% between the primary structures of those peptides, which comprise three apparent regions, an N-terminal loop, a central amphipathic α helix, and a C-terminal anti-parallel β-sheet. The helix is separated from the β-sheet by turn T1 and the two β-sheets by turn T2. In addition to the similar structural topology, the specifics of different insect defensins are diverse. The loop motif shows high variability, especially for DLP4, where the terminal of the loop account for its particular variability. The helix always ends with the R23 residue, followed by a G residue which is involved in a hydrogen bond with the carbonyl group of C3 (Landon et al., 2008). Hydrophobic side chains distributed over the surface present a number of hydrophobic spots, some of which correspond to conservative AA: A1, T2, L5, L6, A15, and A17; others are more specific: P8, A14, and W29 for DLP4, I9, L18, L21, and F22 for Sapecin C; and I11 for Phormicin A. Charged residues at the end of the helix, turns T1 and T2, and the C-terminus form positive areas. Variable and conserved regions were also displayed (Figure 1B), based on these regions, peptides ID1∼ID3 were designed by substituting cysteines in one pair of disulfide bonds with A residue; ID4∼ID7 were changed at the conservative sites with AA of similar properties; ID8∼ID26 were generated with varied charges through a basic or acidic AA replacement; ID27∼ID30 were designed by changing hydrophobicity while keeping charge unchanged (Table 1 and Supplementary Table S1).

**TABLE 1:** Physicochemical parameters of parental and designed peptides.

Peptide	Sequences	MW^a^	Net charge	Hydrophobicity	A1^b^	A2^c^
DLP4	ATCDLLSPFKVGHAACAAHCIARGKRGGWCDKRAVCNCRK	4269.05	6	0.354	16	16
ID1	AT**A**DLLSPFKVGHAACAAHCIARGKRGGW**A**DKRAVCNCRK	4206.93	6	0.292	–	–
ID2	ATCDLLSPFKVGHAA**A**AAHCIARGKRGGWCDKRAV**A**NCRK	4206.93	6	0.292	–	–
ID3	ATCDLLSPFKVGHAACAAH**A**IARGKRGGWCDKRAVCN**A**RK	4206.93	6	0.292	–	–
ID4	ATCD**I**LSPFKVGHAACAAHCIARGKRGGWCDKRAVCNCRK	4269.05	6	0.356	32	ND
ID5	ATCDL**I**SPFKVGHAACAAHCIARGKRGGWCDKRAVCNCRK	4269.05	6	0.356	16	ND
ID6	ATCDLL**T**PFKVGHAACAAHCIARGKRGGWCDKRAVCNCRK	4283.08	6	0.361	8	16
ID7	ATCDLLSPFKVGHAACAAHCIARGKR**S**GWCDKRAVCNCRK	4299.08	6	0.353	–	–
ID8	ATCDLLS**K**FKVGHAACAAHCIARGKRGGWCDKRAVCNCRK	4300.11	7	0.311	32	ND
ID9	ATCDLLSPFKV**K**HAACAAHCIARGKRGGWCDKRAVCNCRK	4340.17	7	0.329	16	ND
ID10	ATCDLLSPFKVGHAACAAHCIARGKRGGWC**N**KRAVCNCRK	4268.07	7	0.358	16	ND
ID11	ATCDLLSPF**G**VGHAACAAHCIARGKRGGWCDKRAVCNCRK	4197.93	5	0.379	16	ND
ID12	ATCDLLSPFKVGHAACAAHCIARG**N**RGGWCDKRAVCNCRK	4254.98	5	0.364	–	–
ID13	ATCDLLSPFKVGHAACAAHCIARGKRGGWCD**G**RAVCNCRK	4197.93	5	0.379	4	4
ID14	ATCDLLSPFKVGHAACAAHCIARGKRGGWCDKRAVCNCR**N**	4254.98	5	0.364	–	–
ID15	ATCDLLSPF**G**VGHAACAAHCIARG**N**RGGWCDKRAVCNCRK	4183.86	4	0.388	–	–
ID16	ATCDLLSPF**G**VGHAACAAHCIARGKRGGWCD**G**RAVCNCRK	4126.81	4	0.403	8	16
ID17	ATCDLLSPF**G**VGHAACAAHCIARGKRGGWCDKRAVCNCR**N**	4183.86	4	0.388	16	64
ID18	ATCDLLSPFKVGHAACAAHCIARG**N**RGGWCD**G**RAVCNCRK	4183.86	4	0.388	16	ND
ID19	ATCDLLSPFKVGHAACAAHCIARG**N**RGGWCDKRAVCNCR**N**	4240.91	4	0.373	–	–
ID20	ATCDLLSPFKVGHAACAAHCIARGKRGGWCD**G**RAVCNCR**N**	4183.86	4	0.388	16	ND
ID21	ATCDLLSPF**G**VGHAACAAHCIARG**N**RGGWCD**G**RAVCNCRK	4112.74	3	0.413	–	–
ID22	ATCDLLSPFKVGHAACAAHCIARG**N**RGGWCD**G**RAVCNCR**N**	4169.79	3	0.398	–	–
ID23	ATCDLLSPFKVGHAACAAHCIARGKRGGWCD**D**RAVCNCR**N**	4241.89	3	0.369	–	–
ID24	ATCDLLS**K**FKV**K**HAACAAHCIARGKRGGWCDKRAVCNCRK	4371.23	8	0.286	–	–
ID25	ATCDLLSPFKV**K**HAACAAHCIARGKRGGWC**N**KRAVCNCRK	4339.19	8	0.333	–	–
ID26	ATCDLLS**K**FKVGHAACAAHCIARGKRGGWC**N**KRAVCNCRK	4299.12	8	0.315	–	–
ID27	ATCDLLSPFKVGHAACA**L**HCIARGKRGGWCDKRAVCNCRK	4311.13	6	0.388	8	8
ID28	ATCDLLSPFKVGHAACA**V**HCIARGKRGGWCDKRAVCNCRK	4297.1	6	0.377	8	16
ID29	ATCDLLSPFKVGHAACAAHC**L**ARGKRGGWCDKRAVCNCRK	4269.05	6	0.351	64	128
ID30	ATCDLLSPFKVGHAACAAHCIARGKRGGWCDKRA**I**CNCRK	4283.08	6	0.368	16	32

*Changed amino acids are shown in bold and underlined. ^a^Molecular weight (Da). ^b^ Activities (MICs, μg/mL) of peptides were tested against S. aureus ATCC 43300; inactive peptides are indicated by “–,” tested by drop-diffusion assay. ^c^ Activities (MICs, μg/mL) of peptides were tested against S. aureus CVCC 546; inactive peptides are indicated by “–,” tested by drop-diffusion assay; ND: not determined.*

The peptides listed in Table 1 were inserted into plasmids and successfully expressed in *P. pastoris* X-33. The production of peptide ID13 was performed in a 5-L fermenter with total protein of 761 mg/L and biomass of 385 g/L at 120 h by induction (Figure 1C). A molecular weight of 4197.01 Da was shown by MALDI-TOF/TOF MS (Figure 1D), which was consistent with the theoretical molecular mass of 4197.93 Da of ID13 (Table 1).

### Bioavailability of ID13

#### Antibacterial Activity

The MICs of DLP4 and its analogs were tested preferentially against *S. aureus* ATCC 43300, among them, peptide ID13 (with the MIC of 4 μg/mL) exerted the most potent antibacterial activity against *S. aureus* ATCC 43300, and the MICs of other 29 derivatives were 8∼64 μg/mL (Table 1). The MICs of peptide ID13 were further tested against other Gram-positive bacteria and Gram-negative bacteria. The result showed that ID13 (with MICs of 4∼8 μg/mL) had higher activity than DLP4 (with MICs of 16∼64 μg/mL) against *S. aureus* CVCC 546, *S. epidermidis* ATCC 12228, *S. pneumoniae* CVCC 2350, and *S. suis* CVCC 3928 (Table 2). However, similar to DLP4, ID13 had no activity against Gram-negative bacteria.

**TABLE 2:** MICs of the designed peptides against pathogenic strains.

Species and strains	MICs					
	ID13		DLP4		Vancomycin	
	μM	μg/mL	μM	μg/mL	μM	μg/mL
**Gram-positive bacteria**						
*Staphylococcus aureus* CVCC 546	0.95	4	3.75	16	0.67	1
*S. epidermidis* ATCC 12228	1.91	8	14.99	64	0.67	1
*Streptococcus pneumoniae* CVCC 2350	0.95	4	7.50	32	0.34	0.5
*S. suis* CVCC 3928	0.95	4	3.75	16	0.17	0.25
**Gram-negative bacteria**						
*Escherichia coli* ATCC 25922	>30.50	>128	>29.98	>128	86.15	128
*E. coli* K88	>30.50	>128	>29.98	>128	43.08	64
*Salmonella pullorum* CVCC 533	>30.50	>128	>29.98	>128	86.15	128
*S. enteritidis* CVCC 3377	>30.50	>128	>29.98	>128	86.15	128

#### Hemolysis and Cytotoxicity of Peptide ID13

**FIGURE 2:**
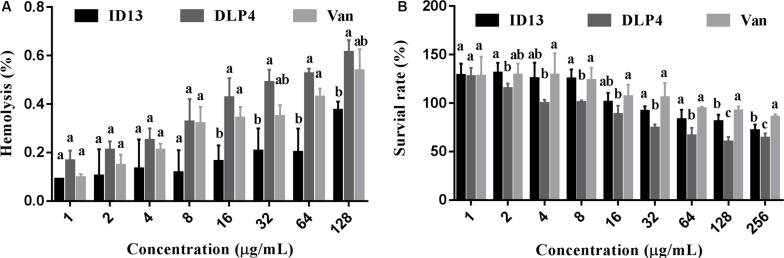
**(A)** Hemolytic activities of ID13, DLP4, and vancomycin (Van) against mouse erythrocytes (*n* = 3). **(B)** Cytotoxicity of ID13, DLP4, and vancomycin (Van) against RAW 264.7 (*n* = 4). Mean values in the same concentration with different lowercase letters indicate a significant difference (*p* < 0.05).

As shown in Figure 2, the hemolysis of ID13 against mouse erythrocytes was 0.38% at 128 μg/mL, lower than that of DLP4 (0.61%) and vancomycin (0.54%). The cell viability of ID13 against mouse macrophages RAW 264.7 was 71.4% at 256 μg/mL, higher than that of DLP4 (63.8%), but lower than that of vancomycin (85.8%). The results indicated that ID13 had lower hemolysis and cytotoxicity than DLP4.

### Structure Determination of Peptide ID13

#### Disulfide Bonds of Peptide ID13

To locate the disulfide bonds in ID13, the cysteine-containing peptide pairs were obtained by thermolytic digestion, and characterized by MALDI-TOF/TOF MS. As shown in Supplementary Figure S1, the disulfide bond–unreduced peptide fragments ATCDLLSPFK and GGWCDGR showed a m/z of 921.4, suggesting they were connected by Cys3 and Cys30, and VGHAACAAHCIAR and AVCNCR had a m/z of 970.45, indicating they were cross-linked by Cys13 and Cys23, and Cys20 and Cys38. The results agreed with those predicted from the Def-AcAA (Figure 1A).

#### CD of Peptide ID13

**FIGURE 3:**
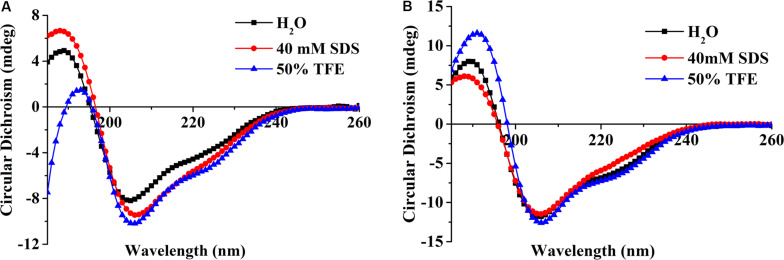
CD spectra of the peptide ID13 **(A)** and DLP4 **(B)** in H_2_O, 40 mM SDS, or 50% TFE.

The secondary structure of the peptide was measured in the presence of H_2_O, 50% TFE, or 40 mM SDS which were used to simulate aqueous, microbial membrane and hydrophobic conditions, respectively (Tan et al., 2019). CD spectra showed peptide ID13 (Figure 3A) as well as DLP4 (Figure 3B) possessed a positive peak between 185 and 195 nm followed by two negative peaks at approximately 208 and 222 nm, indicating an αβ structure in all three environments. Compared with DLP4, the CD peaks of ID13 decreased in H_2_O and 50% TFE and increased in 40 mM SDS. It suggested that ID13 tended to form α-helix in microbial membrane (40 mM SDS) conditions while DLP4 was prone to form α-helix in hydrophobic conditions (50% TFE).

### Anti-*S. aureus* Mechanism of Peptide ID13

#### Effects of ID13 on *S. aureus* Membrane

##### Membrane permeabilization

**FIGURE 4:**
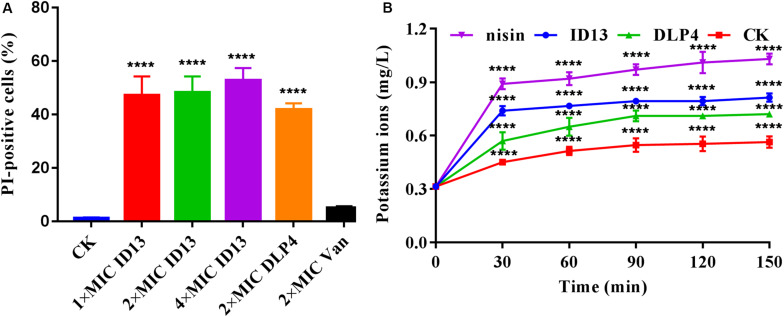
**(A)** Flow cytometric analysis of *S. aureus* CVCC 546. Cells were treated with 1×, 2×, or 4 × MIC ID13, respectively; cells treated with 2 × MIC DLP4 or vancomycin (Van) and left without treatment were used as the positive and negative control, respectively (*n* = 3). **(B)** The extracellular levels of K^+^ released by *S. aureus* CVCC 546 cells treated with peptide ID13, DLP4, and nisin, respectively (*n* = 3). Statistical significance of differences was determined using **(A)** one-way and **(B)** two-way ANOVA followed by Dunnett’s multiple comparison. ****p < 0.0001.

Propidium iodide can penetrate the damaged cell membrane and intercalate into DNA and was utilized to assess the membrane integrity of *S. aureus* CVCC 546 cells. As shown in Figure 4A, in the absence of peptides, 1.18% of the cells showed PI fluorescent signal. When treated with ID13 at 1×, 2×, or 4 × MIC for 0.5 h, the percentage of PI-positive cells were 47.2%, 48.2%, and 52.8%, respectively. By comparison, after being treated with 2 × MIC DLP4 or vancomycin, the percentages of PI-positive cells were 41.8% and 5.1%, respectively. It indicated that ID13 induced more potent damage to *S. aureus* CVCC 546 than its parent peptide DLP4, and the action mode of ID13 and DLP4 may be different from that of vancomycin.

##### K^+^ leakage

To further demonstrate the impacts of peptide ID13 on the cell membrane integrity of *S. aureus* CVCC 546 cells, the levels of K^+^ released from *S. aureus* CVCC 546 cells were determined by ICP-MS. As shown in Figure 4B, in the absence of peptide ID13, the amount of extracellular K^+^ was relatively stable at low level of 0.31∼0.56 mg/L. By comparison, after being treated for 150 min, the levels of released K^+^ from *S. aureus* CVCC 546 were up to 0.81 mg/L by ID13, 0.72 mg/L by DLP4, and 1.03 mg/L by nisin. The results suggested that the destruction of the cell membrane by ID13 is more potent than DLP4, which was consistent with the results of PI-positive cells.

#### Effects of ID13 on *S. aureus* Genomic DNA

##### DNA binding

**FIGURE 5:**
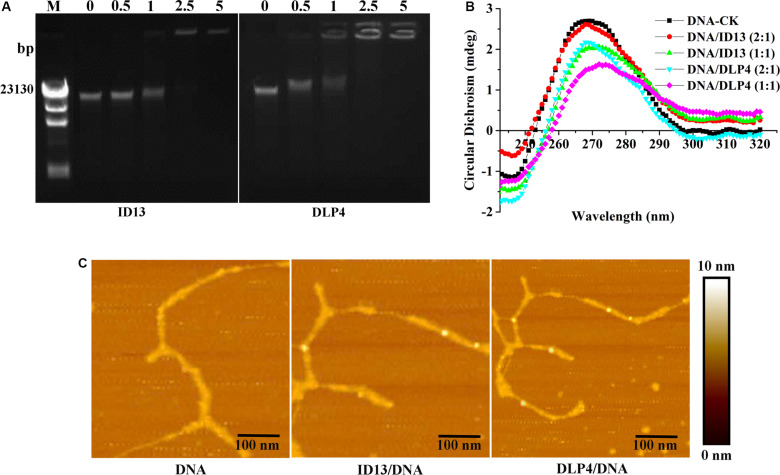
The binding of ID13 and DLP4 to *S. aureus* CVCC 546 gDNA. **(A)** Gel retardation analysis of the binding of ID13 and DLP4 to *S. aureus* CVCC 546 gDNA at peptide/gDNA mass ratios of 0, 0.5, 1, 2.5, and 5, respectively. M: λDNA/Hind III marker. **(B)** Atomic force microscopy (AFM) analysis of the binding of ID13 and DLP4 to *S. aureus* CVCC 546 gDNA. **(C)** CD spectra of *S. aureus* CVCC 546 gDNA at peptide/gDNA mass ratios of 0, 0.5, and 1, respectively.

To probe potential intracellular targets, the DNA gel retardation assay and AFM were performed. As shown in Figure 5A, ID13 began to retard the migration of *S. aureus* CVCC 546 gDNA at the peptide/gDNA mass ratio of 1 while DLP4 did at 0.5. The DNA-binding ability was enhanced with the increase in peptide/gDNA mass ratio. gDNA was completely blocked at ID13/gDNA of 5 and that for DLP4/gDNA at 2.5. Furthermore, the AFM images also showed that DLP4 had a stronger binding ability to gDNA than that of ID13 (Figure 5B), which may ascribe to its more net positive charges (Table 1).

##### CD spectra

Circular dichroism was utilized to estimate the conformation changes of bacterial gDNA caused by ID13. Native bacterial gDNA presents a B conformation, which provides a conservative CD spectrum with a positive band around 270 nm and a negative one around 245 nm (Yao et al., 2018). When exposed to ID13, precipitation in gDNA was observed, the positive band at 270 nm decreased and the negative one at 245 nm gradually disappeared. In contrast, the CD band of DLP4-treated gDNA showed more intense changes (Figure 5C), which may attribute to its increased charge. It indicated that ID13 as well DLP4 can interact with gDNA and destruct its structure.

### Morphological Characterization

**FIGURE 6:**
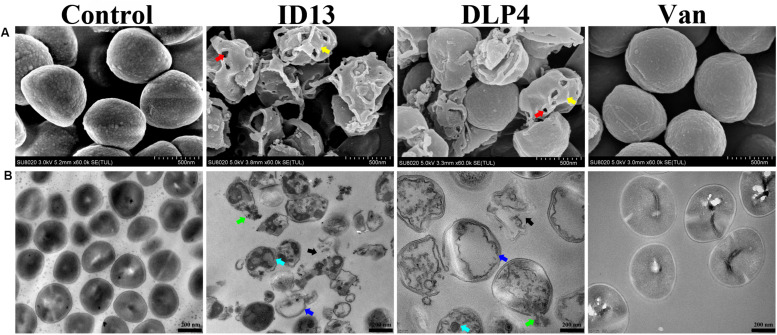
**(A)** SEM and **(B)** TEM micrographs of *S. aureus* CVCC 546 treated with ID13, DLP4, or vancomycin (Van) at 4 × MIC for 2 h or left untreated as control. Red arrow: membrane perforation; yellow arrow: cell shrinkage; green arrow: leakage of cytosol; blue arrow: empty regions; cyan arrow: mesosome-like structure; black arrow: cell rupture.

The microscopic morphology and ultrastructure changes of *S. aureus* CVCC 546 were observed by SEM and TEM. As shown in Figure 6A, compared with the intact control group, the cell surface of peptide ID13-treated *S. aureus* CVCC 546 was significantly changed, with evident cell envelope perforation and deformation. TEM image showed a complete microscopic surface and a dense internal structure of *S. aureus* CVCC 546 cells in the control. In contrast, ID13 significantly damaged *S. aureus* CVCC 546 ultrastructure, including the rupture of cell envelope, the release of intracellular contents, the formation of mesosome-like structure and empty regions, and complete lysis of the cells. Compared to ID13, it seemed DLP4 caused less damage to *S. aureus* CVCC 546 (Figure 6B). Furthermore, there were no obvious morphological changes in the 4 × MIC vancomycin–treated *S. aureus* CVCC 546 cells (Figure 6), indicating different action modes between peptides ID13, DLP4, and vancomycin.

### Pharmacodynamics of Peptide ID13

Based on the attractive mode in which peptide ID13 acts, we investigated its potential as a therapeutic drug through time-kill assay, PAE, and mouse thigh infection model.

#### Time-Kill Assay

**FIGURE 7:**
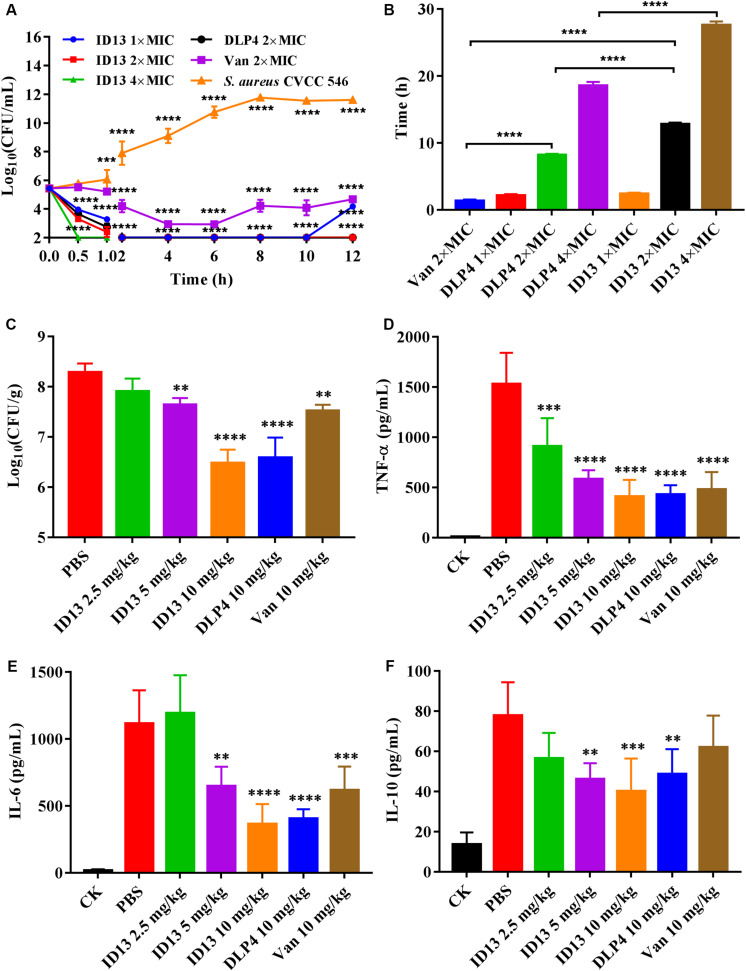
The efficacy of peptide ID13 *in vitro* and *in vivo* with DLP4 and vancomycin (Van) as positive controls. **(A)** Time-kill assay of ID13, DLP4, and vancomycin (Van) against *S. aureus* CVCC 546 (*n* = 3). **(B)** PAEs of ID13, DLP4, and vancomycin (Van) against *S. aureus* CVCC 546 (*n* = 3). **(C)** Single i.p. treatment with ID13, DLP4, and vancomycin (Van) in the mouse thigh infection model (*n* = 6). Effects of ID13, DLP4, and vancomycin (Van) on inflammatory cytokine levels of **(D)** TNF-α, **(E)** IL-6, and **(F)** IL-10. Statistical significance of differences was determined using the two-way ANOVA for **(A)** and one-way ANOVA for **(B–F)**, followed by Dunnett’s multiple comparison. (*) indicates the significance between ID13, DLP4, or vancomycin and PBS. **p < 0.01; ***p < 0.001; ****p < 0.0001.

As shown in Figure 7A, after being treated with 1×, 2×, or 4 × MIC ID13, the CFU counts of *S. aureus* CVCC 546 decreased within 1 h by 2.16, 3.14, and 3.4 log_10_ CFU/mL, respectively, equivalent to a CFU reduction of more than 99.99%. In contrast, for 2 × MIC DLP4 or vancomycin, there was a reduction of 2.7 log_10_ CFU/mL and 0.22 log_10_ CFU/mL, respectively. The results suggested that ID13 had more potent bactericidal activity against *S. aureus* CVCC 546 cells than did DLP4 and vancomycin. Furthermore, the regrowth of bacterial cells was observed after being treated with 2 × MIC vancomycin in the post-incubation time. Note that the common clinical failure of vancomycin in the treatment of *S. aureus* may relate to its the poor bactericidal activity (Sakoulas et al., 2004; Kollef, 2007).

#### PAE

Post-antibiotic effect is an important indicator of medication frequency (Zhanel and Craig, 1994). ID13 produced a longer PAE (12.78 ± 0.28 h) against *S. aureus* CVCC 546 than that of DLP4 (8.20 ± 0.19 h) and vancomycin (1.36 ± 0.20 h) at 2 × MIC. Increasing the concentrations of ID13 significantly prolonged its duration of PAE (Figure 7B).

#### Mouse Thigh Infection Model

The *in vivo* efficacy was then performed in a mouse thigh infection model. At 2 h post-infection with *S. aureus* CVCC 546, ID13 was administered i.p. at single doses ranging from 2.5 to 10 mg/kg. The result showed that ID13 generated a 1.8 log_10_ (CFU/g) reduction in infected thigh muscles at 10 mg/kg, superior to DLP4 with 1.7 log_10_ (CFU/g) and vancomycin with 0.77 log_10_ (CFU/g) (Figure 7C). Moreover, it observed a significant downregulation of serum levels of TNF-α, IL-6, and IL-10 by 1123.30 pg/mL, 750.02 pg/mL, and 37.66 pg/mL, respectively, superior to DLP4 (TNF-α: 1103.30 pg/mL, IL-6: 710.02 pg/mL, and IL-10: 29.06 pg/mL) and vancomycin (TNF-α: 1052.98 pg/mL, IL-6: 497.48 pg/mL, and IL-10: 15.82 pg/mL) at the same concentration (10 mg/kg) (Figures 7D–F).

## Discussion

*Staphylococcus aureus* is disreputable for its potential to get resistance to antibiotics, attracting attention to novel antimicrobial strategies (Chambers and Deleo, 2009; Wenzel et al., 2014). The clinical development of AMPs is currently under evaluation. However, most of these AMPs have low antimicrobial activity and relatively high cytotoxicity (Fjell et al., 2011; Kubicek-Sutherland et al., 2017; Barreto-Santamaria et al., 2019), which limit their therapeutic application[15]. Insect defensins are a group of evolutionarily conserved AMPs with a length distribution between 32 and 52 residues and charges vary from −5 to +8. They share a common CSαβ motif, which have been proved to be valuable structural templates in peptide engineering (Koehbach, 2017). In this work, a naturally occurring sequence DLP4 was used as a template to design more potent and low toxic molecules.

In general, bioactivity of AMPs is strongly associated with their structures; especially the presence of intramolecular disulfide bonds is critical for their conformation and bioactivity (Koehbach, 2017). In our work, peptides ID1–ID3 (with one pair of disulfide bonds blocked by alanine) presented no activity in a drop-diffusion assay (Table 1). Similar results have also been reported for lucifensin and coprisin (Cerovsky et al., 2011; Lee et al., 2014). Studies have shown that truncated defensins at the N-terminal caused decreased antimicrobial activity (Lee et al., 1998; Cerovsky et al., 2011), yet fail to identify single residues that are responsible and highlighting the complexity of sequence-based approaches. In our study, single-conserved AA mutation L5I, L6I, and S7T (with ID4, ID5, and ID6, respectively) in the N-terminal loop had no significant effect on the antibacterial activity. However, the mutant ID7 (with G27S) showed no antibacterial activity, which may be due to the effect of hydroxyl groups of S residues on the anti-parallel sheet conformation (Merkel and Regan, 1998). Additionally, the net positive charges play an important role in influencing the antibacterial activity of AMPs (Toke, 2005); however, the correlation between peptide charge and bioactivity is complex (Huang et al., 2010). In our study, it showed no linear relationship between the charge and activity of peptides (Table 1); however, it presented a threshold (+4∼+7), within which the engineered peptides (ID8∼ID26) showed antimicrobial activity. It has been reported that charged residues (K or R) located at the T1 (AA from 24 to 27) and T2 turns (AA 32 and 33) on the surface of the molecules are particularly important for the activity of insect defensins (Landon et al., 2008); however, in our study, the substitution of K32 in T2 turn with G (ID13) generated a more antimicrobial activity and less toxic molecule. Moreover, amphipathicity is another main parameter responsible for the activity of the engineered peptides (Zelezetsky and Tossi, 2006; Huang et al., 2010). When replacing the hydrophobic AA (A6) of the α-helix wheel with the similar but more hydrophobic AA (L or V) which further improves hydrophobicity, the antimicrobial ability of the designed peptides increased compared to the native peptide. Likewise, the mutation I9L of the α-helix wheel with decreased hydrophobicity resulted in a declined antimicrobial ability (Table 1 and Supplementary Figure S2). Studies showed that hydrophobic residues in the β-sheet replaced by A residue resulted in dramatically decreased biological function of the engineered peptides (Walters et al., 2009; Yang et al., 2009). However, in our study, when the hydrophobic V35 in the β-sheet was replaced by I residue, the activity of peptide ID30 was not affected. Altogether, the CSαβ scaffold is a prerequisite for the activity of the peptide, whereas the activity of the peptides has no linear relationship with charge but increases with the overall hydrophobicity.

The MIC value is a key factor in preliminary screening of candidate peptides, for which ≤16 μg/mL or ≤16 μmol/L has been posed as a requirement in clinical studies (Barreto-Santamaria et al., 2019). In this study, compared with DLP4, ID13 had a lower MIC value (4∼8 μg/mL), hemolytic activity (0.38%), and cytotoxicity (71.4% viability) (Table 2 and Figure 2), which may be related to the different α-helix content between peptide ID13 and DLP4 in bacterial membrane mimicking (40 mM SDS) and neutral membrane conditions (50% TFE) (Figure 3; Yang et al., 2019); the higher α-helix content of peptides indicates the stronger affinity of peptide to cell membranes and thus more potent activity and toxicity (Barreto-Santamaria et al., 2019). An increase in total hydrophobicity in a certain range also increased the antibacterial activity of ID13 (Table 1). The cell envelope is regarded as the initial contact point of most natural AMPs. Then, AMPs can destroy the cell envelope and effectively induce a leakage of cytosol (Huang et al., 2010). It has been proven in our study that ID13 had the ability to destroy the cell envelope and cause K^+^ or other contents to release extracellularly (Figures 4, 6), and then bound to intracellular target gDNA and destroyed its helical structure (Figure 5), which might result in the inhibition of DNA synthesis (Gottschalk et al., 2015). In contrast, DLP4 showed less potent damage to cell membrane than that of ID13 (Figures 4, 6), but it showed stronger binding ability to *S. aureus* gDNA (Figure 5), which might attribute to its less α-helix content in bacterial membrane mimicking conditions and more net positive charges. Other peptides also showed cell membrane damaging abilities to different bacteria (Morgan et al., 2005; Li et al., 2012). The action mode of ID13 and DLP4 is completely different from that of vancomycin which targets the D-Ala-D-Ala terminus of peptidoglycan to inhibit the cell wall biosynthesis (Wang F. et al., 2018).

Pharmacodynamics has been integral to the design of rational drug dosing regimens (Uckun and Qazi, 2019). Our findings showed that ID13 had excellent bactericidal efficiency, a long PAE, and high efficacy in a mouse thigh infection model against *S. aureus* CVCC 546 and was superior to DLP4 or vancomycin (Figure 7). Compared with antibiotics, AMPs is less easily to cause bacterial resistance (Moravej et al., 2018b), and it is likely that any changes of bacteria to avoid the AMPs attack are also to grievously influence the proliferation of bacteria (Andersson et al., 2016). All these mean a lower dose and less frequency in administration, thereby potentially reducing treatment costs, drug exposure, and drug resistance. Above all, ID13 presents excellent properties and potency for biomedical applications.

In conclusion, a naturally occurring sequence DLP4 was used as a starting template for rational design of a novel class of “CSαβ AMPs.” Of these peptides, ID13 showed the best antibacterial activity and reduced cytotoxicity and could penetrate and destroy the cell membrane of *S. aureus* CVCC 546, resulting in an increase in K^+^ leakage. After being treated with ID13, perforated, wrinkled, and collapsed *S. aureus* cells were observed by SEM and TEM. Additionally, ID13 showed potent bactericidal efficiency, a prolonged PAE, and high efficacy in a mouse thigh infection model and was superior to DLP4 or vancomycin. These results suggest that ID13 may be a novel promising antimicrobial candidate to treat infectious diseases caused by *S. aureu*s.

## Data Availability Statement

All datasets generated for this study are included in the article/Supplementary Material.

## Ethics Statement

The animal study was reviewed and approved by the Animal Care and Use Committee of the Feed Research Institute of Chinese Academy of Agricultural Sciences.

## Author Contributions

BL, RM, DT, XW, and JW conceived and designed the experiments. BL carried out all the experiments. BL, NY, ZL, and ZW prepared partial materials in the laboratory. DT, XW, RM, and JW contributed to writing. JW contributed to funding acquisition. YH contributed materials and reagents.

## Conflict of Interest

The authors declare that the research was conducted in the absence of any commercial or financial relationships that could be construed as a potential conflict of interest.

## Footnotes

1 http://aps.unmc.edu/AP
