# Supplementary material for: An Enhanced Variant Designed From DLP4 Cationic Peptide Against Staphylococcus aureus CVCC 546
Source: Front Microbiol. 2020 Jun 5;11:1057. doi: 10.3389/fmicb.2020.01057 (PMC7291858; doi:10.3389/fmicb.2020.01057)
Supplement: Supplementary file 1 [file Data_Sheet_1.docx]

Supplementary Material

## Supplementary Table

**Table S1.** The design principles and purpose of peptide

| Peptides | Design principles for peptide | Purpose of peptide design |
| --- | --- | --- |
| ID1~ID3 | Substituting cysteines in one pair of disulfide bonds with alanines | Roles of cysteines for the activity of peptides |
| ID4~ID7 | Changed the conservative sites with amino acid of similar properties | Roles of conservative sites for the activity of peptides |
| ID8~ID26 | Changing charges | Roles of charge for the activity of peptides |
| ID27~ID30 | Changing hydrophobicity while keeping charges unchanged | Roles of hydrophobicity for the activity of peptides |

## Supplementary Figures

**
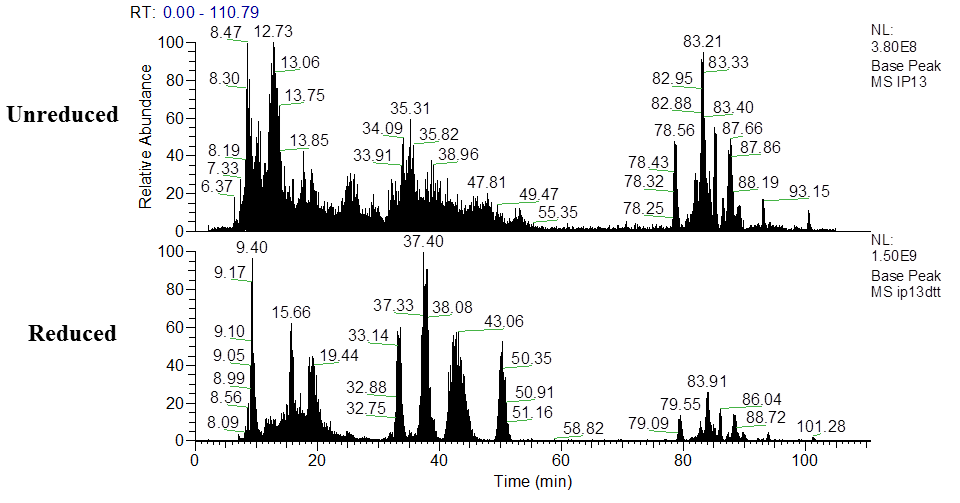
**

**Figure S1A** Total ion current (TIC) diagram of ID13 samples with unreduced or reduced disulfide bonds.


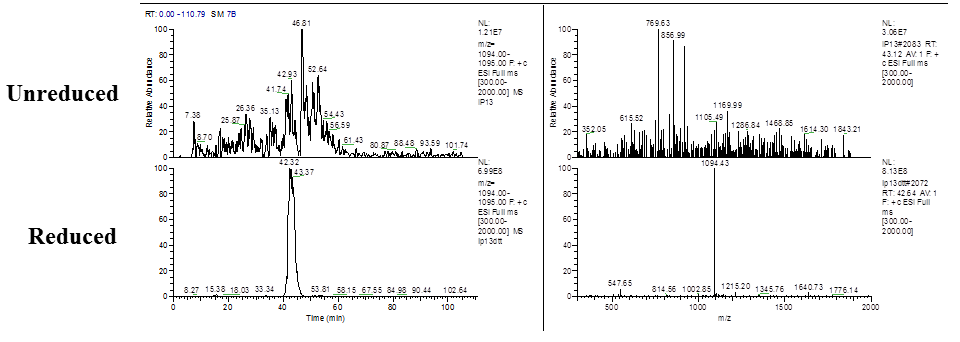


**Figure S1B.** TIC and primary mass spectra of ATCDLLSPFK with unreduced or reduced disulfide bonds.


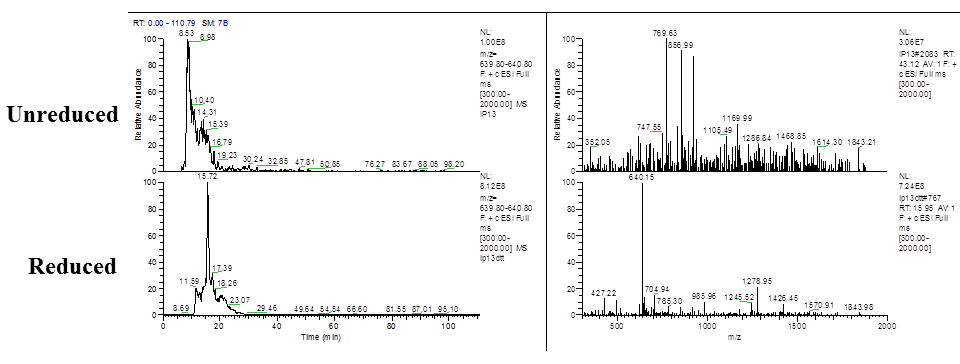


**Figure S1C.** TIC and primary mass spectra of VGHAACAAHCIAR with unreduced or reduced disulfide bonds.


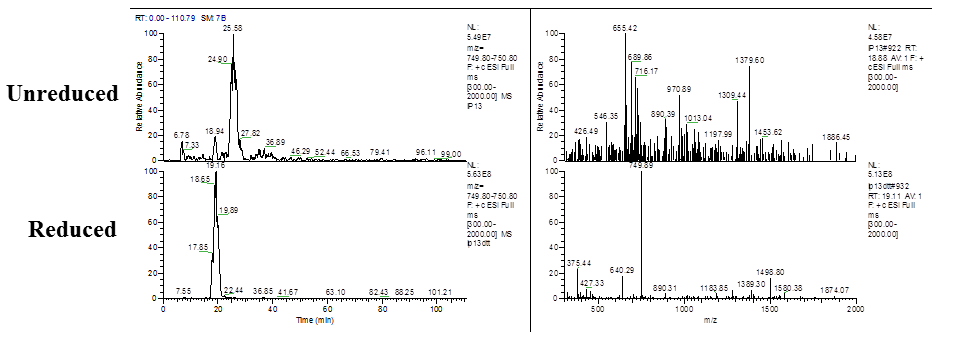


**Figure S1D.** TIC and primary mass spectra of GGWCDGR with unreduced or reduced disulfide bonds.


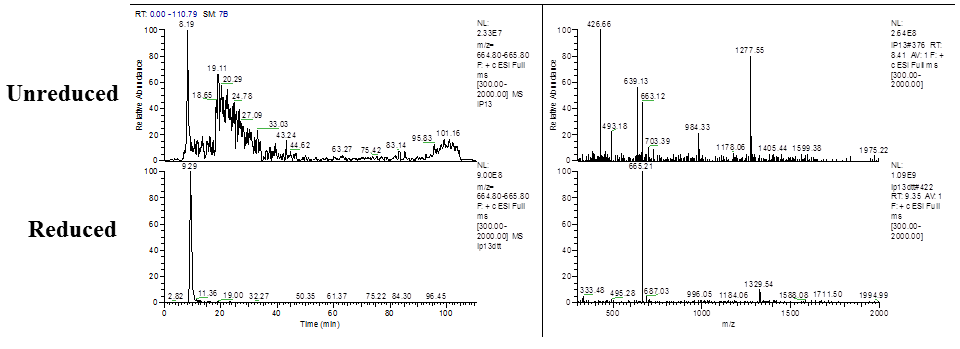


**Figure S1E.** TIC and primary mass spectra of AVCNCR with unreduced or reduced disulfide bonds.


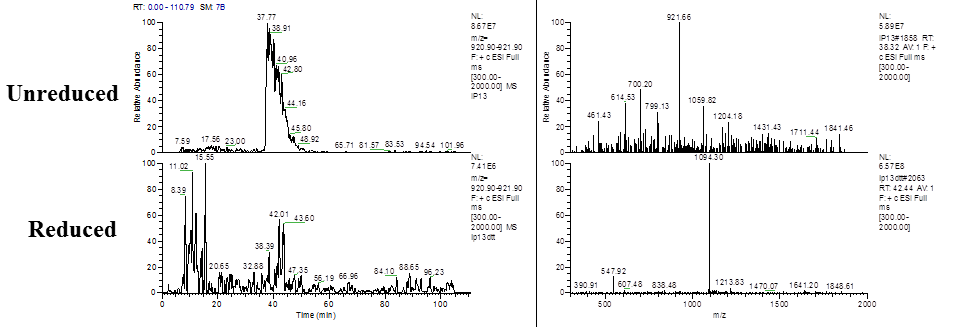


**Figure S1F.** TIC and primary mass spectra of ATCDLLSPFK and GGWCDGR with unreduced or reduced disulfide bonds.


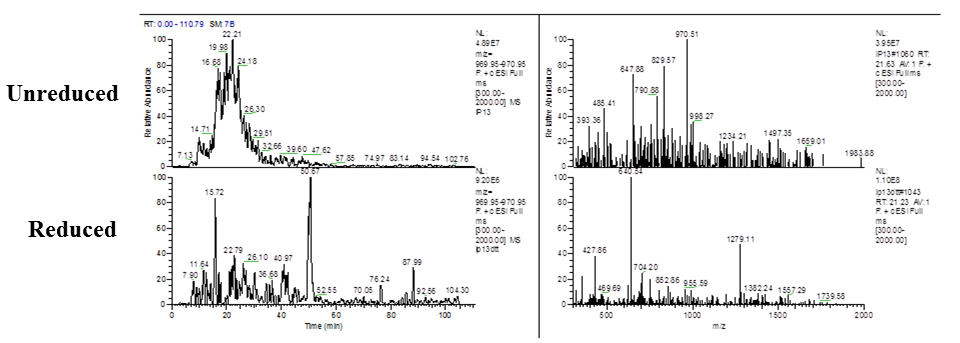


**Figure S1G.** TIC and primary mass spectra of VGHAACAAHCIAR and AVCNCR with unreduced or reduced disulfide bonds.

**
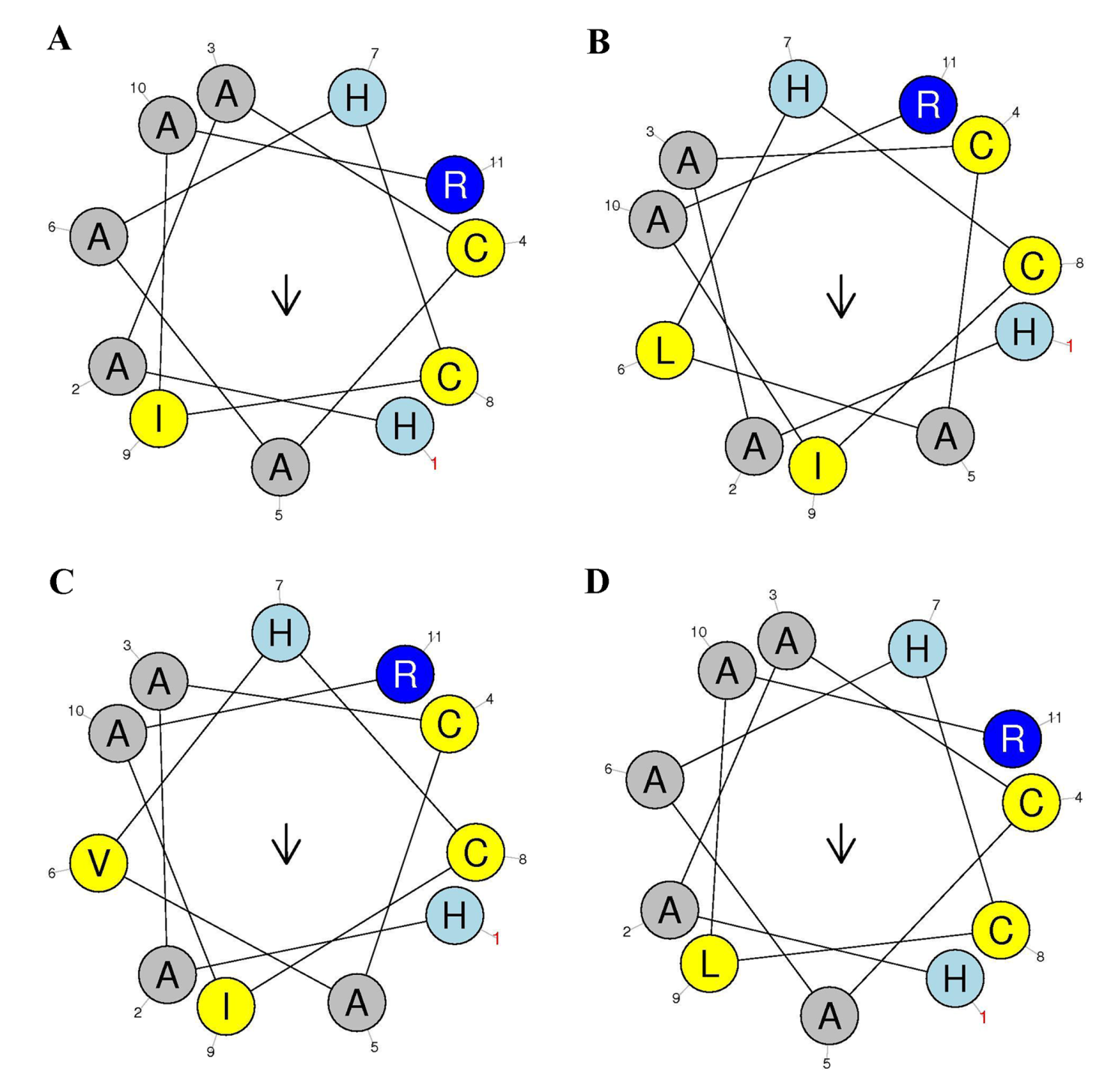
**

**Figure S2.** Helical wheels of the α-helix motif of peptides **(A)** DLP4, **(B)** ID28, **(C)** ID29 and **(D)** ID30, respectively. The number represents the position of amino acids on the helical wheel. The colors indicate the residues with nonpolar, polar uncharged, or basic property.

.
